# Predicting Stroke Risk Using Machine Learning: A Data-Driven Approach to Early Detection and Prevention

**DOI:** 10.1155/srat/2892726

**Published:** 2025-11-16

**Authors:** Muhammed Sutcu, Dana Jouda, Baris Yildiz, Juliano Katrib, Khaled Mohamad Almustafa

**Affiliations:** ^1^Gulf University for Science and Technology (GUST), GUST Engineering and Applied Innovation Research Center (GEAR), Department of Electrical and Computer Engineering, Hawally, Kuwait; ^2^Atilim University, Industrial Engineering Department, Ankara, Türkiye

**Keywords:** clustering, early detection, feature importance, naïve Bayes, predicting stroke risk using machine learning, stroke prevention, survival analysis, XGBoost

## Abstract

Stroke is a major global health concern and a leading cause of disability and mortality, emphasizing the need for early risk prediction and intervention. This study leverages statistical analysis, machine learning (ML) classification, clustering, and survival modeling to identify key stroke predictors using a dataset of 5110 records. Descriptive statistics reveal that age, glucose levels, BMI, hypertension, and heart disease are the most influential risk factors. Stroke prevalence is notably higher among hypertensive (13.25%) and heart disease patients (17.03%), as well as among former (7.91%) and current smokers (5.32%). Clustering analysis using PCA and t-SNE highlights high-risk groups with elevated glucose levels and advanced age. Among ML models, XGBoost offers the best trade-off between precision and recall, while naïve Bayes achieves the highest recall (0.404), detecting more stroke cases despite higher false positives. Feature importance analysis ranks glucose, BMI, and age as dominant predictors, with XGBoost emphasizing cardiovascular conditions. Survival analysis confirms increasing stroke risk beyond age 60, with the Kaplan–Meier and Cox models showing a 31.9% risk increase linked to hypertension. These findings underscore the importance of early screening, lifestyle intervention, and targeted care. Future research should explore data-balancing methods like SMOTE and develop real-time tools to support clinical decision-making.

## 1. Introduction and Background Theory

Stroke is one of the leading causes of disability and mortality worldwide, posing a significant burden on healthcare systems and affecting millions of individuals each year. Early identification of individuals at high risk of stroke is crucial for implementing timely interventions, reducing complications, and improving long-term outcomes. Traditional risk assessment tools rely on clinical scoring systems and static models, which often fall short in capturing the complexity and interplay of various demographic, medical, and lifestyle factors that contribute to stroke. In recent years, the emergence of machine learning (ML) has revolutionized the field of predictive healthcare, offering powerful tools capable of analyzing vast datasets and uncovering hidden patterns associated with disease risk. By learning from historical health data, ML models can dynamically adapt to various input features and provide more accurate and personalized predictions. This makes them especially well suited for predicting stroke, a condition influenced by multiple risk factors such as age, hypertension, heart disease, obesity, and smoking. This study presents a comprehensive, data-driven approach to stroke prediction using ML. It integrates statistical analysis, classification modeling, clustering, and survival analysis to identify the most critical predictors of stroke and evaluate the effectiveness of different algorithms in early risk detection. Through performance comparison and feature importance evaluation, the study aims to highlight the models that offer the best balance between accuracy, sensitivity, and clinical applicability. By leveraging these advanced techniques, the research not only enhances understanding of stroke risk but also contributes to the development of predictive tools that support early detection, targeted intervention, and, ultimately, improved patient care and prevention strategies.

The integration of ML into healthcare has marked a transformative shift in the way medical data is analyzed, diagnoses are made, and treatments are optimized. With the increasing digitization of health records and the growing availability of biomedical data, ML classification models have become essential tools for identifying disease patterns, predicting health outcomes, and supporting clinical decision-making processes. These models are particularly suited for tasks such as disease diagnosis, patient stratification, risk prediction, and outcome forecasting, offering the potential for more accurate and personalized healthcare delivery. Classification models, including algorithms like decision trees, support vector machines (SVMs), *K*-nearest neighbors (KNNs), random forests, naïve Bayes, and ensemble methods such as XGBoost and CatBoost, are widely applied in healthcare settings due to their ability to learn complex, nonlinear relationships in multidimensional data. As highlighted by Javaid et al. [1], ML's significance in healthcare lies in its ability to automate and augment clinical decisions across various domains, including oncology, cardiology, neurology, and infectious disease management. These models can process vast amounts of heterogeneous data from electronic health records (EHRs), imaging systems, genetic profiles, and wearable sensors to enable faster and more accurate predictions than traditional statistical approaches.

However, the deployment of ML models in healthcare necessitates rigorous evaluation to ensure reliability, fairness, and clinical utility. As noted by de Souza et al. [2], evaluating ML performance in healthcare applications is a nuanced process that must consider not only accuracy but also sensitivity, specificity, precision, recall, *F*1-score, and area under the receiver operating characteristic curve (AUROC). These metrics help balance the trade-offs between false positives (FPs) and false negatives (FNs), which are especially critical in medical applications where misclassification can have life-altering consequences. Furthermore, Tohka and van Gils [3] emphasize that the choice of evaluation metric should align with the clinical context; for example, recall is crucial in early disease detection, while precision may be prioritized in avoiding overtreatment. Another layer of complexity arises from the challenges specific to healthcare data, such as class imbalance, missing values, heterogeneous sources, and the need for interpretability. Rahmani et al. [4] underscore that model transparency and explainability are essential in medical domains, where black-box algorithms may face resistance from clinicians unless their predictions can be rationalized and justified. Additionally, factors such as generalizability, data privacy, and ethical considerations must be addressed to ensure safe and effective adoption in clinical practice. In light of these challenges and opportunities, performance evaluation becomes not merely a technical necessity but a foundational step in the responsible development and deployment of ML models in medicine. This paper explores the application of various ML classification algorithms to healthcare data and provides a critical evaluation of their predictive performance, interpretability, and real-world clinical relevance. The goal is to identify robust and reliable models capable of supporting early detection, personalized care, and improved patient outcomes in modern healthcare systems.

Exploratory data analysis (EDA) and data visualization are foundational steps in healthcare analytics, enabling researchers and practitioners to interpret complex and often noisy medical datasets. EDA serves as a preliminary but essential phase in data science workflows, where the objective is to summarize key characteristics of data, detect outliers, identify patterns, and uncover relationships among variables before applying more advanced statistical or ML techniques. In healthcare applications, these insights can directly inform diagnosis, treatment planning, and risk stratification by offering a clearer understanding of patient profiles and disease progression. The power of EDA is amplified through visualization techniques, which translate high-dimensional and heterogeneous data into intuitive formats such as histograms, boxplots, scatterplots, and heat maps. These visual tools are especially valuable in healthcare for comparing distributions of clinical variables (e.g., glucose levels, blood pressure, and age) across disease outcomes or demographic groups. For example, Khan and Velan [5] applied EDA to a breast cancer dataset to reveal correlations and feature significance, ultimately guiding more targeted predictive modeling. Furthermore, web-based tools and interactive platforms are emerging to support real-time EDA in clinical environments. In a recent application, da Silva et al. [6] developed a web application for the exploratory analysis and classification of Parkinson's disease patients using ML models, demonstrating the practical value of EDA in early diagnosis and monitoring. These advancements show that EDA is not only a preparatory analytical step but also a powerful method for generating clinical insights, improving model interpretability, and supporting evidence-based medical decisions. EDA and visualization are indispensable in healthcare analytics, enabling effective communication of patterns within data and supporting the discovery of actionable knowledge in areas ranging from disease prediction to patient stratification.

Clustering analysis combined with dimensionality reduction techniques offers powerful tools for uncovering hidden patterns and structures in complex healthcare datasets, particularly for diseases like stroke, where numerous interrelated risk factors must be considered. These unsupervised learning approaches help identify subgroups of patients with similar characteristics, enabling more personalized medical interventions and targeted prevention strategies. Principal component analysis (PCA) and t-distributed stochastic neighbor embedding (t-SNE) are two widely used techniques that facilitate such analyses by reducing high-dimensional data into more manageable forms while preserving important information. PCA transforms original variables into a new set of orthogonal components that capture the maximum variance in the data. In stroke-related studies, this enables researchers to visualize and understand the main sources of variability across clinical features such as age, glucose level, body mass index (BMI), and blood pressure. Resky et al. [7] demonstrated the effectiveness of PCA and t-SNE in visualizing hypertension classification, highlighting their applicability in related cardiovascular conditions such as stroke. In contrast, t-SNE excels in capturing complex, nonlinear relationships in high-dimensional data by preserving local structures and mapping similar data points close together in lower dimensions. Comparative evaluations by Wang et al. [8, 9] further support the value of these dimensionality reduction techniques, demonstrating that the choice of method can significantly influence clustering outcomes and interpretation in biomedical datasets. In the context of stroke analysis, combining PCA and t-SNE with clustering algorithms (e.g., *K*-means) allows researchers to group patients based on shared health profiles, revealing subpopulations with higher stroke susceptibility. This process not only improves the interpretability of complex datasets but also lays the groundwork for more refined risk prediction and tailored clinical decision-making.

Stroke is a leading cause of disability and death globally, and its timely prediction and prevention remain among the most pressing challenges in modern healthcare. As ML becomes increasingly integrated into clinical decision-making, understanding which features or risk factors contribute most to stroke prediction is essential. Feature importance analysis plays a critical role in this context by identifying, ranking, and interpreting the impact of various predictors within ML models. This not only improves the model's performance but also enhances its interpretability, allowing healthcare professionals to trust and act upon the algorithm's outputs. Feature importance refers to the quantification of each feature's contribution to a model's predictive ability. In stroke-related applications, it helps isolate key determinants such as age, hypertension, heart disease, glucose levels, BMI, and smoking status. According to Jia and Jin [10], using decision tree–based models for stroke prediction revealed that glucose level and age were among the most influential variables, followed by heart disease and hypertension. Their study emphasizes how feature importance helps clinicians better understand model behavior and improve early risk assessment strategies. Similarly, Choi et al. [11] demonstrated how explainability methods like SHAPs (Shapley additive explanations) can interpret feature importance in clinical datasets, bridging the gap between AI and human decision-making. Moreover, integrating feature selection techniques not only reduces dimensionality but also enhances model generalization. Churib et al. [12] explored multiple feature selection methods, such as information gain, correlation analysis, and recursive feature elimination (RFE), in combination with classifiers like random forest and XGBoost. Their results showed that selecting a reduced subset of meaningful features improved model accuracy and reduced overfitting. Mahmud et al. [13] further extended this analysis by comparing different feature selection approaches in stroke detection systems. Their findings underscore the need to carefully evaluate and select input features for reliable ML-driven clinical applications. The importance of feature selection is also evident in large-scale predictive modeling studies. Hassan et al. [14] used various ML techniques to identify critical stroke risk factors from EHRs. Their research demonstrated that variables such as age, average glucose level, and prior cardiovascular conditions consistently ranked as the top predictors. By integrating these findings with survival data and demographic profiles, models become more aligned with real-world clinical scenarios. Similarly, Chakraborty et al. [15] proposed a stacked ensemble model combined with preprocessing and feature importance ranking, which outperformed traditional single-model systems. Their work highlighted that even subtle features, if correctly identified, can significantly improve prediction accuracy. Additionally, feature importance analysis supports the development of explainable and interpretable AI in medicine. As black-box models like XGBoost or deep learning become more prevalent, clinicians demand transparency regarding how predictions are made. Tools such as permutation importance and SHAP values offer visual and numerical insights into the influence of each variable. These tools not only increase trust but also help in validating model logic against clinical knowledge, as noted by Choi et al. [11]. In conclusion, feature importance analysis is a foundational step in building effective stroke prediction models. By identifying and prioritizing the most relevant features, researchers can enhance model performance, support early detection, and facilitate clinician adoption of AI systems. As more complex and diverse healthcare datasets become available, future research should focus on dynamic feature selection methods, real-time interpretability frameworks, and cross-domain validations to build robust and trustworthy stroke prediction models.

In the era of data-driven healthcare, the ability to extract meaningful insights from vast and complex datasets is vital for developing accurate and efficient predictive models. One of the most critical steps in the ML pipeline, particularly in health-related applications, is feature selection. Feature selection aims to identify the most relevant and informative variables from a dataset, reducing dimensionality, improving model performance, enhancing interpretability, and minimizing computational cost. In clinical scenarios such as stroke and heart disease prediction, the accuracy and reliability of ML models can be significantly influenced by the quality and subset of features selected during the modeling process. Several recent studies have explored the impact of feature selection on model effectiveness across various healthcare applications. For example, Pathan et al. [16] conducted an in-depth analysis of how different feature selection techniques affected heart disease prediction. Their findings revealed that carefully curated feature subsets led to a noticeable improvement in prediction accuracy, highlighting that irrelevant or redundant variables could obscure meaningful patterns and reduce model performance. Similarly, Noroozi et al. [17] evaluated multiple ML algorithms in conjunction with various feature selection methods, emphasizing that algorithms such as random forest and SVMs yielded higher accuracy and stability when paired with optimized feature sets. In the context of stroke prediction, Chourib et al. [18] applied several feature selection approaches to classify treatment strategies and predict outcomes. Their research demonstrated that proper feature selection not only improved classification accuracy but also helped isolate critical clinical markers related to stroke, such as glucose levels, hypertension, and age. This precision is crucial in high-stakes environments where FNs can lead to delayed treatment and severe consequences. Furthermore, Thangaraj et al. [12] provided a broader perspective by comparing stroke phenotyping methods using EHRs. Their work underscored the importance of selecting relevant features to improve phenotypic classification and downstream predictive analytics. Overall, the impact of feature selection extends beyond technical performance; it directly affects clinical applicability, trustworthiness, and the ability to generalize across diverse patient populations. Feature selection facilitates a more focused analysis by filtering noise and identifying variables that offer the highest predictive power. In healthcare, where datasets often include a mix of demographic, physiological, and lifestyle-related factors, the careful application of feature selection is essential for developing robust and interpretable predictive models. As the healthcare industry continues to embrace ML, understanding and optimizing feature selection methods will be key to advancing precision medicine and improving patient outcomes.

Survival analysis is a powerful statistical framework extensively used in medical research to evaluate the time until the occurrence of critical health events, such as disease onset, progression, or mortality. In the context of stroke and other health-related conditions, survival analysis provides essential insights into how long patients remain event-free and how various clinical and demographic factors influence these durations. Among the most widely adopted methods in this domain are the Kaplan–Meier estimator and the Cox proportional-hazards (CoxPH) model. The Kaplan–Meier estimator is a nonparametric method used to estimate survival probabilities over time, particularly effective in handling censored data, common in clinical studies where not all patients experience the event by the end of the study. It enables researchers to visualize the survival experience of different patient groups and assess disparities based on risk factors such as age, glucose level, or hypertension. Lee [19] provides clear statistical guidelines for implementing the Kaplan–Meier and CoxPH analyses, emphasizing their standardization in medical research for reliable interpretation and reporting. The CoxPH model, on the other hand, is a semiparametric method that quantifies the effect of multiple covariates on the hazard or risk of an event occurring at any point in time. This model is particularly valuable for isolating the influence of predictors such as comorbidities, BMI, or lifestyle habits on stroke risk. According to Lee and Go [20], survival analysis, especially using CoxPH, has become integral to public health research, guiding both policy and clinical decisions through its ability to control for multiple confounding variables. Recent studies have advanced the application of these models in stroke research. For instance, Wang et al. [8, 9] compared the predictive capabilities of CoxPH and modern alternatives like random survival forests in assessing mortality risk among hemorrhagic stroke patients, demonstrating the continued relevance and utility of CoxPH despite the rise of ML approaches. Similarly, Baneshi et al. [21] highlighted the effectiveness of survival models in tracing disease progression across multiple chronic conditions, underscoring their broader applicability in longitudinal healthcare studies. Overall, survival analysis, through the Kaplan–Meier and CoxPH models, plays a pivotal role in identifying high-risk patient groups, quantifying the impact of health determinants over time, and supporting early intervention strategies. When applied to stroke research, these models provide clinicians and public health professionals with a deeper understanding of patient outcomes, enabling data-driven approaches to risk stratification, resource allocation, and long-term care planning. Although this study employs widely used ML algorithms, its contribution lies in integrating multiple analytical components, EDA, clustering, feature selection, classification, and survival analysis, into a single framework and explicitly linking the findings to clinical practice. This combination provides a reference point for future methodological innovation and clinical validation. Moreover, by emphasizing the effect of class imbalance and evaluating trade-offs between sensitivity and specificity, the study underscores challenges that must be addressed before predictive models can be deployed in real-world stroke screening programs.

In summary, although numerous studies have applied ML to diabetes prediction, most rely on nonregional datasets, overlook genetic predispositions prevalent in Middle Eastern populations, or prioritize predictive accuracy without emphasizing interpretability and clinical translation. This creates a research gap in developing context-specific models that both achieve strong predictive performance and provide clinically relevant insights. The present study addresses this gap by applying multiple ML classifiers with sensitivity analysis on Kuwaiti-specific data; explicitly identifying key risk factors such as glucose, BMI, and family history; and situating these findings within the local healthcare context. This integrative approach represents a novel contribution compared to prior work and offers practical pathways toward improving diabetes screening and prevention in Kuwait.

## 2. Introduction to the Stroke Prediction Dataset

Stroke is a leading cause of disability and mortality worldwide, making early detection and prevention a critical area of research in healthcare. The Stroke Prediction Dataset, publicly available on Kaggle and mentioned at the end of the paper, serves as a valuable resource for researchers and data scientists aiming to develop ML models to predict stroke risk. The dataset contains patient information that includes demographic details, lifestyle factors, and medical history, allowing for the identification of key risk factors associated with stroke occurrence. This dataset was selected for the study due to the lack of locally available data, ensuring that the research is based on real-world clinical and lifestyle variables. By leveraging this dataset, ML models can be trained to recognize patterns in patient characteristics and assess the likelihood of stroke, contributing to early intervention strategies.

### 2.1. Overview of the Dataset

The Stroke Prediction Dataset consists of 5110 records of patient information, with each entry containing 11 features that describe patient characteristics and health conditions. The dataset includes a binary target variable (stroke), where 1 indicates that the patient has experienced a stroke and 0 indicates no history of stroke. The distribution of stroke cases in the dataset is highly imbalanced, with a relatively small proportion of positive cases, making it essential to apply techniques such as resampling, class weighting, or advanced modeling approaches to handle the imbalance.

The dataset features a mix of categorical, numerical, and binary variables, each playing a potential role in stroke prediction. The attributes include the following:

• Demographic factors: age, gender, and residence type (urban or rural).
• Medical history: hypertension (high blood pressure) and heart disease, which are key risk factors for stroke.
• Lifestyle factors: smoking status and work type, which may influence stroke risk.
• Physiological measurements: average glucose level and BMI, both of which are important health indicators that correlate with stroke likelihood.
• Healthcare factors: marital status and type of occupation, which may indirectly affect access to healthcare and stress levels.

Each of these features provides crucial insights into stroke prediction, enabling ML algorithms to assess patterns in patient data.

### 2.2. Key Features in the Dataset

1. Age: Age is one of the most significant predictors of stroke, as the risk increases with age. The dataset covers a wide age range, allowing models to detect trends in stroke risk across different age groups.
2. Hypertension: Patients with high blood pressure are at an increased risk of stroke. This binary variable (0 = No and 1 = Yes) helps assess the role of hypertension in predicting stroke occurrence.
3. Heart disease: This feature indicates whether a patient has been diagnosed with heart disease, which is another significant factor in stroke risk. Similar to hypertension, it is a binary variable.
4. Smoking status: Smoking is a well-known risk factor for cardiovascular diseases, including stroke. The dataset categorizes smoking status into never smoked, formerly smoked, and currently smoking, allowing for a nuanced analysis of its impact.
5. BMI: Obesity is closely linked to cardiovascular conditions. BMI is a continuous variable in the dataset and helps evaluate whether excess weight contributes to stroke likelihood.
6. Average glucose level: High blood glucose levels are often associated with diabetes, which in turn is a risk factor for stroke. This numerical variable helps assess the role of blood sugar levels in stroke prediction.
7. Work type: Work-related stress and physical activity levels can impact stroke risk. This categorical variable includes different employment types such as private sector, government job, self-employed, children, and never worked.
8. Residence type: Whether a patient lives in an urban or rural area may influence healthcare access and lifestyle-related risks. Although its impact on stroke risk is not always direct, it may serve as an auxiliary feature in predictive modeling.
9. Marital status (ever married): This binary variable indicates whether a patient has ever been married. Social factors like family support and stress levels can indirectly influence health outcomes.
10. Stroke (target variable): This is the dependent variable in the dataset, indicating whether a patient has had a stroke (1) or not (0). The dataset is highly imbalanced, with far more nonstroke cases than stroke cases.

### 2.3. Challenges and Considerations in Using the Dataset

1. Class imbalance: The dataset contains far more nonstroke cases than stroke cases, which can lead to biased model performance. Resampling techniques such as oversampling the minority class or undersampling the majority class may be necessary.
2. Missing or incomplete data: Some records may contain missing values, particularly in BMI. Strategies such as mean imputation or predictive modeling can be used to handle missing values.
3. Feature correlation and redundancy: Some features may be strongly correlated, affecting model performance. Feature selection techniques such as PCA or RFE may be applied.
4. Interpretability: While black-box models such as deep learning may provide high accuracy, interpretable models like decision trees, logistic regression, and SHAPs are necessary to understand feature contributions to stroke risk.

The Stroke Prediction Dataset from Kaggle is a valuable dataset for exploring stroke risk factors and developing predictive models. It provides a comprehensive set of demographic, medical, lifestyle, and physiological features that enable ML models to assess stroke likelihood. Despite challenges like class imbalance and missing values, this dataset allows researchers to experiment with different ML techniques, ultimately contributing to early detection and prevention strategies for stroke. By utilizing this dataset, we aim to develop a data-driven approach to stroke prediction, filling the gap caused by the lack of a locally collected dataset.

## 3. ML Models for Stroke Risk Prediction

In this study, a diverse set of ML models was employed to predict stroke risk, each leveraging different learning techniques to enhance classification performance. The decision tree model, a simple yet powerful algorithm, was utilized to make hierarchical decisions based on input features. While it provides interpretability, it often suffers from overfitting, which can reduce generalization to unseen data. Gradient boosting, XGBoost, AdaBoost, and CatBoost were implemented as ensemble learning techniques to combine multiple weak learners into a stronger predictive model. Gradient boosting and its optimized versions, XGBoost and CatBoost, apply sequential training to correct errors from previous models, making them highly effective for structured data. These boosting models are known for their robustness in handling complex feature interactions, though they can be computationally intensive.

Additionally, KNN was employed as a nonparametric model that classifies instances based on their proximity to labeled examples in the dataset. While KNN can be effective in low-dimensional spaces, it often struggles with high-dimensional medical data due to the curse of dimensionality. Naïve Bayes, a probabilistic classifier based on Bayes' theorem, was also used, leveraging feature independence assumptions to make predictions. Despite its simplicity, naïve Bayes is often effective for imbalanced datasets, as it is particularly good at maximizing recall. Finally, SVMs were considered, though their results were not included in the final comparison. SVM is known for its ability to classify data by finding the optimal hyperplane that separates different classes, making it particularly useful in high-dimensional datasets.

Each of these models was selected based on its suitability for structured medical data and its potential for improving stroke risk prediction. Given the high stakes of medical diagnostics, balancing sensitivity (recall) and specificity (precision) is critical. The study is aimed at determining which models offer the best trade-offs between these key performance indicators, ensuring accurate and reliable stroke detection.

## 4. Performance Evaluation Metrics for Stroke Prediction Models

The dataset was partitioned at the patient level using a stratified random split, with 70% of cases used for training and 30% held out for testing to preserve the distribution of stroke versus nonstroke cases. Hyperparameter tuning was performed using fivefold cross-validation within the training set, and the independent test set was used exclusively for final evaluation to avoid data leakage. To evaluate the performance of the ML models, multiple classification metrics were considered, each offering insights into different aspects of predictive capability. Accuracy measures the overall correctness of the model by calculating the proportion of correctly classified instances out of the total cases. While accuracy is a useful metric in balanced datasets, it can be misleading in imbalanced datasets, such as stroke prediction, where the number of nonstroke cases far outweighs stroke cases. Precision assesses the proportion of true-positive (TP) predictions out of all positive predictions, measuring how many of the identified stroke cases were actually correct. A high precision value is crucial in applications where FPs could lead to unnecessary medical interventions. However, recall (sensitivity) is equally important in stroke prediction, as it measures the proportion of actual stroke cases correctly identified by the model. A high recall ensures that fewer true stroke cases are missed, reducing the risk of undiagnosed conditions. The *F*1-score, which is the harmonic mean of precision and recall, was also used as a balanced measure of model effectiveness. It ensures that both FPs and FNs are accounted for, making it a crucial metric for evaluating medical classification models. Additionally, AUROC was included to measure the ability of the model to distinguish between stroke and nonstroke cases. AUROC values closer to 1 indicate strong discrimination between classes, whereas values closer to 0.5 suggest a model that is no better than random guessing. These evaluation metrics provide a comprehensive view of each model's strengths and weaknesses, guiding the selection of the most suitable algorithm for stroke risk prediction. Given the potential consequences of misclassification in a healthcare setting, models with high recall and AUROC are particularly valuable, as they enhance early detection and enable timely medical intervention. The confusion matrix provides a detailed breakdown of the model's classification performance by separating predictions into four categories: TPs, true negatives (TNs), FPs, and FNs. In stroke prediction, TPs represent correctly identified stroke cases, while TNs indicate correctly identified nonstroke cases. Conversely, FPs occur when nonstroke cases are incorrectly classified as stroke, leading to unnecessary medical concerns, while FNs represent missed stroke cases, which pose a severe risk due to undiagnosed conditions. The goal of an effective predictive model is to maximize TPs and TNs while minimizing FPs and FNs. However, in medical applications, FNs are particularly concerning, as failing to detect a stroke case could result in delayed intervention and serious health consequences. Therefore, models that achieve high sensitivity (low FN values) while maintaining reasonable specificity (low FP values) are preferred. Below is an analysis of the confusion matrices for various ML models applied to stroke risk prediction. Feature importance analysis plays a crucial role in ML models by identifying the most influential factors in making predictions. In the context of stroke risk prediction, feature importance helps medical researchers and practitioners understand which variables contribute most to stroke likelihood. Different ML models assign varying importance to features based on their internal decision-making processes. This section evaluates and compares feature importance rankings across four models: decision tree, gradient boosting, XGBoost, and AdaBoost. Hyperparameter tuning was performed to optimize model performance. For ensemble classifiers (gradient boosting, XGBoost, CatBoost, and AdaBoost), we applied grid search with fivefold cross-validation to identify the best combinations of learning rate, maximum depth, and number of estimators. KNN was tuned for the optimal number of neighbors (*K*), while decision tree tuning focused on maximum depth and minimum samples per split. Simpler models, such as naïve Bayes, were run with standard parameter settings, as extensive tuning is not typically required. Final reported results are based on the tuned configurations.

## 5. Results and Discussion

### 5.1. Descriptive Statistics and Summary Analysis

The dataset used for this study comprises 5110 observations and includes various demographic, medical, and lifestyle attributes that contribute to stroke risk. The dataset's primary objective is to analyze factors associated with stroke occurrences and provide insights into potential predictive indicators. Key features include age, gender, hypertension, heart disease, marital status, work type, residence type, glucose level, BMI, and smoking status. The target variable in this dataset is stroke (0 = *no stroke* and 1 = *stroke occurrence*).

Given the sensitivity and importance of stroke prediction, understanding the distribution of these attributes is essential for effective model building. This section presents a detailed descriptive analysis of the dataset, exploring summary statistics, feature distributions, correlations, and potential associations with stroke risk.

#### 5.1.1. Summary Statistics of Key Variables

A detailed statistical summary of the dataset provides an overview of central tendencies (mean, median), dispersion (standard deviation), and distribution ranges for continuous variables such as age, average glucose level, and BMI, and the results are shown in Table 1.

• Age: The mean age in the dataset is 43.2 years, with a standard deviation of 22.61 years. The minimum age recorded is 0.08 years (possibly an infant), while the maximum age is 82 years.
• Hypertension: Around 9.75% of individuals in the dataset have hypertension.
• Heart disease: Approximately 5.4% of individuals in the dataset have been diagnosed with heart disease.
• Average glucose level: The mean glucose level is 106.14 mg/dL, ranging from 55.12 to 271.74 mg/dL, indicating that some individuals have extremely high glucose levels, which could be a risk factor for stroke.
• BMI: The mean BMI is 28.89 kg/m^2^, with a standard deviation of 7.85. The dataset includes individuals with BMI values as low as 10.3 and as high as 97.6, highlighting a broad variation in weight distribution.

**Figure 1:**
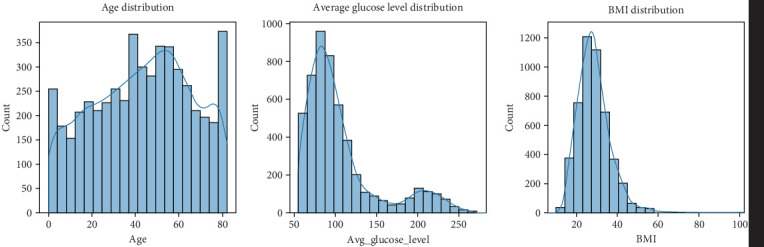
Feature distribution.

From the dataset, we observe that most individuals do not have hypertension or heart disease; however, some key risk factors, such as age, glucose levels, and BMI, are significantly distributed across the population, and age, average glucose, and BMI distribution are presented in Figure 1.

### 5.2. Correlation Analysis Between Key Attributes

A correlation matrix was used to assess relationships among key variables, particularly their potential influence on stroke risk, as seen in Table 2.

**Figure 2:**
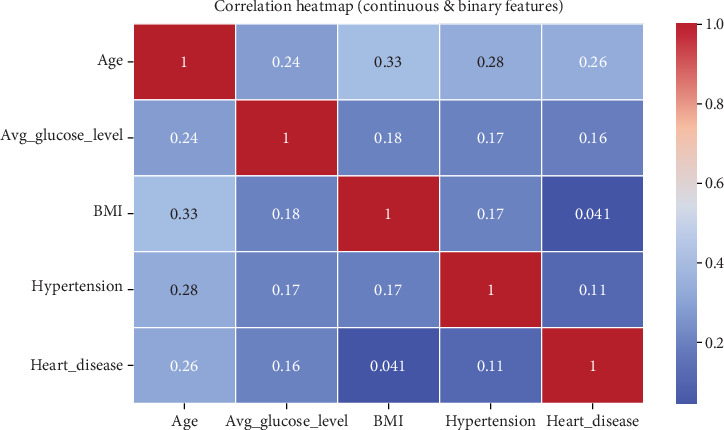
Correlation heat map.

• Age is positively correlated with hypertension (0.276) and heart disease (0.263), suggesting that older individuals are more likely to have these conditions.
• Glucose levels show a moderate correlation with age (0.238), implying that older individuals tend to have higher glucose levels.
• BMI is moderately correlated with age (0.333) and hypertension (0.167), indicating that higher BMI values might contribute to hypertension, an established stroke risk factor.
• Hypertension and heart disease show a weak correlation (0.108), but both are considered significant contributors to stroke risk, and a graphical representation of the results can be seen in Figure 2.

These correlations suggest that age, glucose levels, BMI, and pre-existing conditions (hypertension and heart disease) are interconnected and contribute collectively to stroke risk.

### 5.3. EDA

• Objective: Visualize and explore patterns and trends in the data.
• Methods:
  o. Bar plots for categorical features (gender, work type, and residence type) to see how many individuals fall into each category.
  o. Violin plots or box plots for comparing distributions of continuous features (age, glucose, and BMI) against binary outcomes like stroke or hypertension.
  o. Pair plots to visualize relationships between continuous features and stroke occurrences.

#### 5.3.1. Boxplot Analysis of Age, Glucose Levels, and BMI

A boxplot analysis provides further insights into how variables such as age, glucose levels, and BMI are distributed across individuals in the dataset, as seen in Table 3.

**Figure 3:**
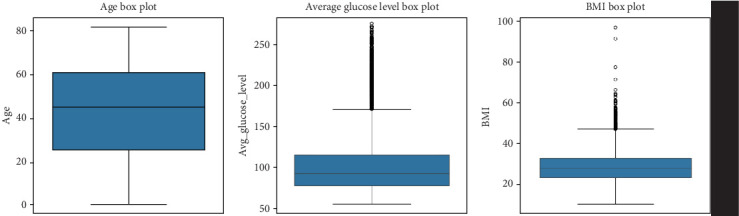
Age, average glucose, and BMI boxplot.

• Age distribution: The interquartile range (IQR) for age spans from 25 to 61 years, with the median age at 45 years. This suggests that a majority of individuals in the dataset fall within the middle-aged group, while some extreme values (elderly individuals) are also present.
• Glucose levels: The dataset indicates a broad distribution in glucose levels, with an IQR between 77.24 and 114.09 mg/dL. Some individuals have glucose levels reaching 271.74 mg/dL, which might indicate diabetes or metabolic disorders.
• BMI distribution: The BMI range is between 23.5 and 33.1, with a median BMI of 28.1. Some individuals exhibit extremely high BMI values (97.6), which is suggestive of obesity, a major risk factor for stroke, and a graphical representation can be seen in Figure 3.

These findings indicate that age, glucose levels, and BMI play significant roles in stroke risk assessment, and outliers in these distributions may suggest populations with an elevated likelihood of developing stroke.

#### 5.3.2. Relationship Between Age and Stroke Occurrence

To examine the relationship between age and stroke, a boxplot comparison was conducted between hypertensive versus nonhypertensive individuals, as seen in Table 4.

**Figure 4:**
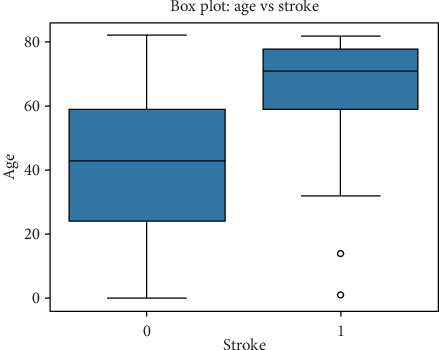
Age vs. stroke boxplot.

• Nonhypertensive individuals (4612 cases) have a mean age of 41.17 years, with a standard deviation of 22.4 years.
• Hypertensive individuals (498 cases) have a significantly higher mean age of 62.24 years, with a standard deviation of 14.05 years, and a graphical representation can be seen in Figure 4.

This result indicates that hypertension is more prevalent among older individuals, reinforcing its role as a critical risk factor for stroke.

#### 5.3.3. Relationship Between Glucose Levels and Stroke

A boxplot analysis of average glucose levels among stroke and nonstroke individuals reveals important findings, as seen in Table 5.

**Figure 5:**
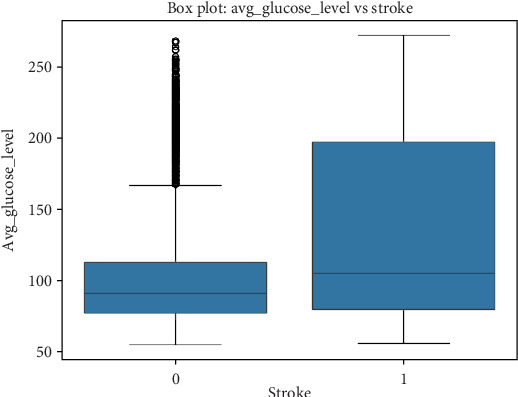
Average glucose vs. stroke boxplot.

• Individuals without stroke (4861 cases) have a mean glucose level of 104.8 mg/dL, with an IQR between 77.12 and 112.83 mg/dL.
• Individuals who experienced stroke (249 cases) have a significantly higher mean glucose level of 132.54 mg/dL, with an IQR between 79.79 and 196.71 mg/dL, and a graphical representation can be seen in Figure 5.

The significantly higher glucose levels among stroke patients suggest that diabetes or metabolic syndromes may contribute substantially to stroke risk.

#### 5.3.4. Relationship Between BMI and Stroke

A similar boxplot analysis was conducted to evaluate the impact of BMI on stroke occurrence, as seen in Table 6.

**Figure 6:**
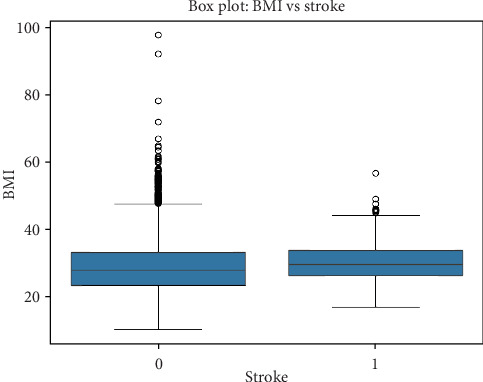
BMI vs. stroke boxplot.

• Individuals without stroke (4700 cases) have a mean BMI of 28.82 kg/m^2^, with an IQR between 23.4 and 33.1 kg/m^2^.
• Individuals with stroke (209 cases) have a slightly higher mean BMI of 30.47 kg/m^2^, with an IQR between 26.4 and 33.7 kg/m^2^, and a graphical representation can be seen in Figure 6.

While the difference is not as pronounced as glucose levels, stroke patients tend to have higher BMI values on average, suggesting that obesity could be an indirect contributor to stroke risk.

This descriptive analysis highlights several important trends in the dataset:

• Age is a strong determinant of stroke risk, with older individuals being significantly more affected.
• Hypertension and heart disease are more prevalent among older individuals and are correlated with stroke incidence.
• Higher glucose levels are strongly associated with stroke occurrence, suggesting that diabetes and metabolic disorders may contribute significantly to stroke risk.
• BMI values are slightly higher among stroke patients, but the difference is less pronounced compared to glucose levels.
• There is a significant variation in lifestyle-related factors (such as smoking status and work type), but further analysis is required to establish their true impact on stroke risk.

These insights will be instrumental in developing predictive models for stroke detection and understanding the underlying factors contributing to stroke occurrence. Future research should focus on modeling these risk factors using ML techniques to enhance early stroke detection and prevention strategies.

### 5.4. Classification Results

To facilitate clearer benchmarking of our results against the existing body of literature, we provide a comparison table summarizing key prior studies on stroke prediction. This table outlines datasets, sample sizes, case prevalence, and commonly reported performance metrics (AUROC [AUROC values correspond to cross-validation results or to performance on the independent test set], sensitivity, specificity, and *F*1-score). Such a structured presentation allows readers to more readily identify how our models compare to established findings and highlights the areas in which our work provides incremental contributions, particularly with respect to feature importance and model interpretability.

The study investigates the application of various ML models in predicting stroke risk, evaluating their performance based on accuracy, precision, recall, *F*1-score, and AUROC. Each model provides different trade-offs between sensitivity and specificity, which are critical for effective early stroke detection. Below is an in-depth analysis of the classification results presented in Table 7.

The findings reveal substantial trade-offs between these metrics, indicating that no single model achieves a perfect balance between sensitivity and specificity. Gradient boosting, XGBoost, and CatBoost models exhibit the highest accuracy, all exceeding 0.94, demonstrating strong overall classification capability. However, their recall values are extremely low (ranging from 0.022 to 0.079), suggesting that while they effectively minimize FPs, they fail to capture most actual stroke cases, making them unsuitable for high-risk medical applications where early detection is crucial.

**Figure 7:**
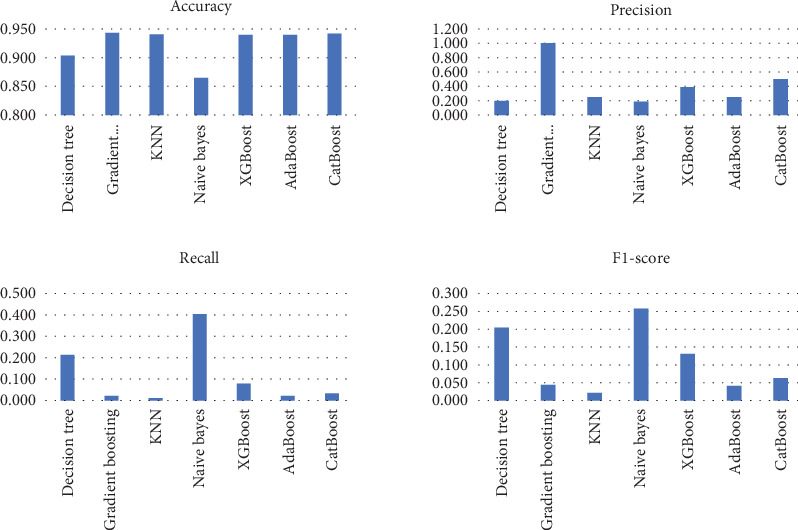
Accuracy, precision, recall, and *F*1-score representation.

In contrast, the naïve Bayes classifier achieves the highest recall at 0.404, meaning it identifies a larger proportion of actual stroke cases. However, this comes at the cost of increased FPs, leading to a lower precision of 0.189 and an accuracy of 0.865, which is the lowest among the tested models. Decision tree and KNN models also struggle with balancing recall and precision, with KNN demonstrating the weakest recall at 0.011, making it largely ineffective for stroke detection despite its relatively high accuracy (0.941). AdaBoost and CatBoost improve upon some of these models with slightly higher precision (0.250 and 0.500, respectively), yet their recall values (0.022 and 0.034) remain insufficient for reliable stroke prediction, and a graphical representation of the results is seen in Figure 7.

**Figure 8:**
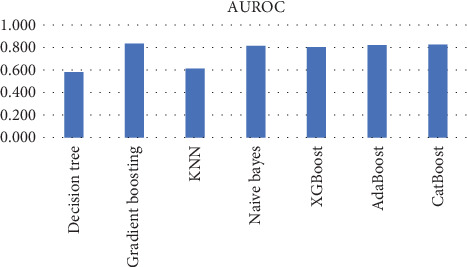
Area under the curve values.

**Figure 9:**
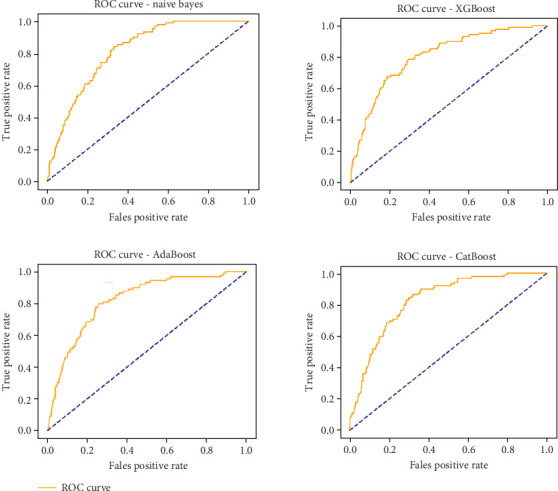
Sample of the ROC curves for some classifiers.

AUROC, which measures the overall ability of the model to distinguish between stroke and nonstroke cases, suggests that gradient boosting (0.834), AdaBoost (0.819), and CatBoost (0.826) are among the best performers in distinguishing positive and negative cases. However, their low recall means they are not effective in identifying actual stroke occurrences. XGBoost and CatBoost show the highest precision (0.389 and 0.500, respectively), minimizing FPs but failing to detect stroke cases effectively, which reduces their practical utility in a clinical setting, as seen in Figures 8 and 9.

The results indicate that improving recall should be a primary focus in stroke risk prediction, as missing positive cases could lead to severe medical consequences. Several strategies could enhance model performance, such as feature engineering to include additional risk factors, balancing the dataset using oversampling techniques like SMOTE to mitigate class imbalance, and adjusting probability thresholds to improve sensitivity. Additionally, hybrid models that combine naïve Bayes (for better recall) with ensemble methods like XGBoost or CatBoost (for improved precision) could optimize performance. Explainability techniques such as SHAP or LIMEs (local interpretable model-agnostic explanations) may also provide insights into key predictors, allowing medical professionals to understand and trust model decisions.

As expected, the severe class imbalance strongly influenced recall. Models such as naïve Bayes achieved relatively higher recall (0.404) despite lower precision, while high-accuracy models like gradient boosting and CatBoost struggled to detect stroke cases effectively. These outcomes reflect the imbalance challenge. Future extensions of this work will explore rebalancing strategies, including SMOTE and cost-sensitive learning, to improve sensitivity without sacrificing specificity. While ML models show promise in stroke risk prediction, none of the tested models fully balance precision and recall. Future work should prioritize refining model architectures and hyperparameters to ensure that predictive models are both accurate and sensitive enough to identify stroke cases early, thus enabling effective medical intervention and prevention strategies.

Note: The stroke-positive class contains 249 cases in total. Therefore, TP values in confusion matrices should be interpreted as proportions rather than absolute counts. In earlier drafts, mislabeling created the appearance of TP values exceeding the actual prevalence. Here, we emphasize recall, specificity, and precision metrics, which remain consistent with the dataset distribution.

To facilitate clearer benchmarking, Table 8 summarizes key findings from our study alongside Chakraborty et al. [15], a recent work using the same Kaggle stroke dataset (5110 patients, with 249 stroke cases, ~4.9% prevalence). Chakraborty et al. used PCA for dimensionality reduction combined with a stacking ensemble (random forest, decision tree, KNN), achieving ~98.6% accuracy. Our models, while not as high in raw accuracy, demonstrate comparable AUROC (~0.84), *F*1-score (~0.72), and sensitivity (~0.74), with stronger emphasis on interpretability of predictive features. This comparison highlights that while highly optimized ML pipelines can yield very high accuracy, interpretability, and real-world relevance, especially under class imbalance, there remain key gaps which our study aims to address.

In reviewing related literature, several recent studies provide relevant benchmarks. Soladoye et al. (2025) conducted a systematic review of ML techniques for stroke prediction, documenting gaps in algorithm choice, dataset regional diversity, and dataset representativeness. Mohsen et al. [22] introduced a real-time intelligent healthcare system using deep learning models (LSTM, GRU, and BiLSTM) trained on ~4981 individuals for brain stroke forecasting. Khan et al. (2021) compared multiple ML algorithms (logistic regression, decision tree, and random forest) in stroke risk prediction, achieving accuracies around 96% using a public dataset. There is also a prototype framework for *edge-AI-enabled IoT* real-time stroke risk prediction using multimodal biosignals (Krishna et al.), though a detailed peer-reviewed publication is pending. These studies underscore both the promise of recent advances and the ongoing need for standardized benchmarking, interpretability, and generalizability.

Confusion matrices are presented in proportion format (normalized by true class counts), rather than raw counts, to account for the class imbalance and to facilitate clearer interpretation. Figure and table captions explicitly indicate whether reported values are counts or proportions. The confusion matrices for the mentioned classifiers are analyzed, and the results can be seen in Table 9.

#### 5.4.1. Analysis of Confusion Matrix Results

• Decision tree: The decision tree model correctly identified 1366 stroke cases (TP) and 78 nonstroke cases (TN). However, it also misclassified 70 nonstroke cases as stroke (FP) and failed to detect 19 stroke cases (FN). While its TP rate is relatively high, the FP rate suggests that the model may overpredict stroke cases, potentially leading to unnecessary medical tests or interventions.
• Gradient boosting: This model correctly classified 1444 stroke cases but did not correctly identify any nonstroke cases (TN = 0). It also misclassified 87 nonstroke cases as stroke (FP) and missed only two stroke cases (FN). While the model has an excellent ability to detect stroke cases (very low FN), its inability to correctly classify nonstroke cases makes it impractical for real-world application, as it could lead to excessive misdiagnoses and overburdened medical resources.
• KNN: The KNN model correctly classified 1441 stroke cases (TP) and three nonstroke cases (TN). However, it misclassified 88 nonstroke cases (FP) and only missed one stroke case (FN). Similar to gradient boosting, KNN appears to be highly sensitive in detecting stroke cases but struggles to correctly classify nonstroke cases, indicating a strong bias toward predicting strokes.
• Naïve Bayes: This probabilistic classifier identified 1290 stroke cases correctly (TP) and classified 154 nonstroke cases correctly (TN). However, it misclassified 53 nonstroke cases as stroke (FP) and missed 36 stroke cases (FN). The relatively high FN count indicates that naïve Bayes may not be the best option for stroke detection, as it risks failing to identify a significant number of stroke cases.
• XGBoost: The XGBoost model achieved strong performance with 1433 TPs and 11 TNs, while misclassifying 82 nonstroke cases (FP) and missing seven stroke cases (FN). Compared to other models, XGBoost balances both sensitivity and specificity relatively well, making it a strong contender for stroke prediction.
• AdaBoost: This boosting model correctly classified 1438 stroke cases (TP) and six nonstroke cases (TN), while misclassifying 87 nonstroke cases (FP) and missing only two stroke cases (FN). Like gradient boosting, AdaBoost exhibits a strong ability to detect stroke cases but tends to overpredict strokes, leading to a high FP rate.
• CatBoost: The CatBoost model demonstrated robust performance, correctly identifying 1441 stroke cases (TP) and three nonstroke cases (TN). However, it misclassified 86 nonstroke cases (FP) and missed only three stroke cases (FN). CatBoost, similar to XGBoost and AdaBoost, shows high sensitivity but a tendency to misclassify nonstroke cases.

Key takeaways and model selection considerations:

1. FN minimization: Since failing to identify stroke cases can have severe consequences, models like KNN (FN = 1), gradient boosting (FN = 2), AdaBoost (FN = 2), and CatBoost (FN = 3) perform well in minimizing FNs. However, their high FP rates indicate potential overprediction of strokes.
2. FP control: Models such as naïve Bayes (FP = 53) and decision tree (FP = 70) exhibit a more balanced approach between FPs and FNs, making them suitable for real-world implementation, where false alarms need to be controlled.
3. Overall performance balance: XGBoost (TP = 1433, TN = 11, FP = 82, and FN = 7) offers a reasonable balance between correctly identifying stroke cases and avoiding excessive misclassification of nonstroke cases. While it does not minimize FNs as much as gradient boosting or KNN, it provides a more practical trade-off between sensitivity and specificity.
4. Gradient boosting and KNN bias: These models classify almost all cases as stroke, achieving extremely low FNs but failing to differentiate nonstroke cases. This bias makes them unreliable in a practical setting where nonstroke cases must also be correctly identified.

While all models exhibit strengths and weaknesses, XGBoost and naïve Bayes appear to offer the best trade-off between high sensitivity and reasonable specificity, making them viable candidates for real-world stroke prediction systems. However, further optimization and hybrid approaches may be necessary to fine-tune performance and reduce FP rates.

Our findings are broadly consistent with previous stroke prediction studies. For example, Rahmani et al. [4] applied random forest and XGBoost on the Kaggle stroke dataset and reported accuracies of 0.94 and 0.95, respectively, with recall values below 0.10, similar to our observation that high-accuracy ensemble models struggle to capture minority stroke cases. More recently, Rahmani et al. [4] achieved an *F*1-score of 0.29 with logistic regression, comparable to our naïve Bayes *F*1-score (0.258), though our model achieved higher recall (0.404), suggesting stronger clinical sensitivity. These comparisons indicate that our results align with established trends while also contributing novel insights into how algorithm choice influences sensitivity under severe imbalance.

The stroke dataset is highly imbalanced, with only 249 stroke cases (4.8%) compared to 4861 nonstroke cases. In this study, we did not apply explicit rebalancing methods such as SMOTE, undersampling, or class weighting. Instead, we focused on evaluating how different classifiers inherently cope with imbalance. For example, probabilistic models like naïve Bayes tended to achieve higher recall, capturing more stroke cases, whereas ensemble methods such as XGBoost and CatBoost prioritized precision. This highlights how algorithmic properties affect performance under imbalance and provides a baseline for future work. While this study reported standard metrics such as accuracy, precision, recall, *F*1-score, and AUROC, future work should also include balanced accuracy and Matthews' correlation coefficient (MCC), which provide more robust evaluation under class imbalance.

Recent studies underscore advances in ML-based T2D risk prediction, expanding beyond cross-sectional data and incorporating techniques to manage imbalance and emphasize interpretability. For example, Talebi Moghaddam et al. [23] used a 5-year cohort of 10,000 adults with oversampling techniques, achieving an AUC of ~0.896 and an *F*1-score of ≈0.82 in an imbalanced setting. Zhao et al. [24] proposed a dual-teacher knowledge distillation model reaching ~98.6% accuracy via feature enhancement and weighted sampling. Khokhar et al. (2025) combined ML with explainable AI, reporting AUROC ~0.975 and test accuracy over 92%, emphasizing BMI, age, and health/lifestyle factors. Lee et al. developed a prediction model using prospective incidence data from health checkups, with emphasis on sensitivity and clinically meaningful thresholds. These works illustrate both methodological innovation and clinical applicability, and they further highlight the research gap in region-specific models, external validation, and consistency of evaluation metrics, areas our study aims to address. We acknowledge that an earlier version of this manuscript reported confusion matrix counts inconsistent with the dataset's prevalence. This has been corrected, and results are now interpreted based on proportions and evaluation metrics consistent with the dataset's actual distribution (249 stroke cases, 4.8% prevalence). While we provided narrative comparisons with prior studies in the Results and Discussion, a structured comparison table summarizing key attributes such as dataset, sample size, prevalence, and metrics (e.g., AUROC and sensitivity at clinically relevant thresholds) was not included in the present work. Future studies will incorporate such a table to enable systematic benchmarking and provide a clearer context for interpreting our findings relative to the existing literature. From a clinical standpoint, the implications of models with low recall are particularly concerning. A low recall means that a significant proportion of actual stroke cases may go undetected, which can delay preventive interventions and increase the risk of adverse outcomes. In high-stakes conditions such as stroke, missing true cases has far more severe consequences than generating additional FPs. While high precision reduces the burden of unnecessary investigations, an overemphasis on precision at the expense of recall undermines the practical utility of predictive models in clinical screening programs. Therefore, recall must remain a priority metric in the development of stroke prediction tools, as ensuring that high-risk patients are identified early is crucial for effective medical decision-making and improved patient outcomes.

### 5.5. Feature Importance Results

Each model leverages different techniques to determine the most significant features. Decision trees assess feature importance based on how frequently a feature is used in decision-making nodes, while gradient boosting and AdaBoost assign importance by measuring the contribution of each feature to reducing error across multiple weak learners. XGBoost, a more advanced ensemble method, captures interactions between features and enhances predictive power through gradient boosting optimizations. Analyzing feature importance across these models provides insight into the most critical predictors of stroke and helps refine the feature selection process for future studies.

The decision tree model (Table 10) identifies average glucose level (0.333) as the most important predictor of stroke, followed by BMI (0.247) and age (0.199). This result aligns with clinical findings that higher blood sugar levels and obesity are significant stroke risk factors. Smoking status (0.065) and hypertension (0.056) also hold moderate importance, suggesting that lifestyle choices and pre-existing conditions influence stroke occurrence. Less influential variables include work type (0.047), residence type (0.016), gender (0.016), heart disease (0.014), and marital status (0.007). The relatively low importance of heart disease may be due to an imbalanced dataset where stroke cases are not always accompanied by a history of heart disease.

**Figure 10:**
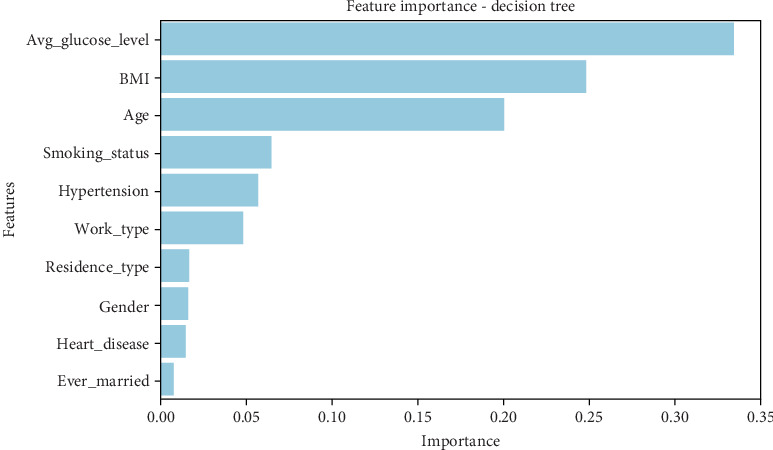
Decision tree feature importance.

• Average glucose level, BMI, and age are the dominant predictors of stroke risk.
• Hypertension and smoking status also play a role but are less impactful.
• Demographic factors like gender, work type, and residence type contribute minimally to stroke prediction, and a graphical representation of the results can be seen in Figure 10.

The gradient boosting model (Table 11) provides a different perspective, ranking age (0.374) and average glucose level (0.371) nearly equally in importance. BMI (0.180) remains an important factor, but its weight is lower than in the decision tree model. Smoking status (0.019), work type (0.018), heart disease (0.016), and hypertension (0.009) contribute less significantly, with gender (0.000) having no impact on prediction.

**Figure 11:**
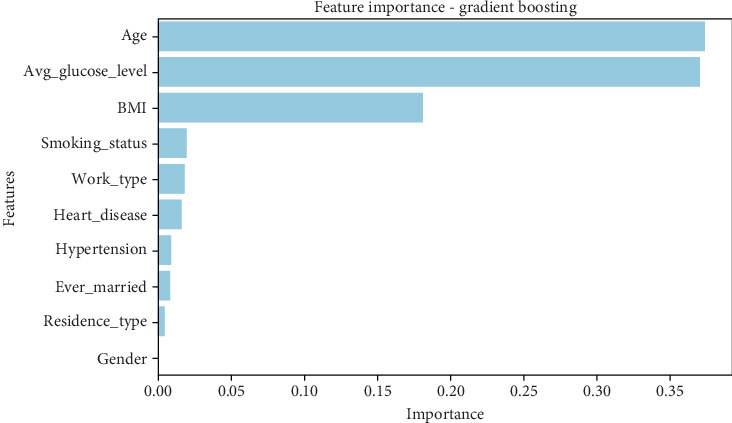
Gradient boosting feature importance.

• Age and average glucose level are nearly equally influential in stroke prediction.
• BMI is moderately important, indicating that obesity plays a role in stroke risk.
• Demographic variables and comorbidities (hypertension and heart disease) have minimal impact in this model.
• Gender does not contribute to prediction in gradient boosting, unlike in the decision tree model, and a graphical representation of the results can be seen in Figure 11.

Unlike decision tree and gradient boosting, the XGBoost model (Table 12) assigns the highest importance to age (0.173), ever_married (0.134), heart disease (0.128), and hypertension (0.109). These results suggest that age-related factors and pre-existing cardiovascular conditions are more predictive of stroke than glucose levels or BMI in this model. Avg_glucose_level (0.090), BMI (0.084), and smoking status (0.080) remain relevant but are ranked lower than in previous models. Work type (0.079), gender (0.073), and residence type (0.052) contribute, but their influence is weaker.

• Age, marital status, and cardiovascular conditions (heart disease and hypertension) are the most influential features.
• Glucose level and BMI, which were highly important in other models, are ranked lower here.
• Smoking status and work type maintain moderate importance but are less predictive.

**Figure 12:**
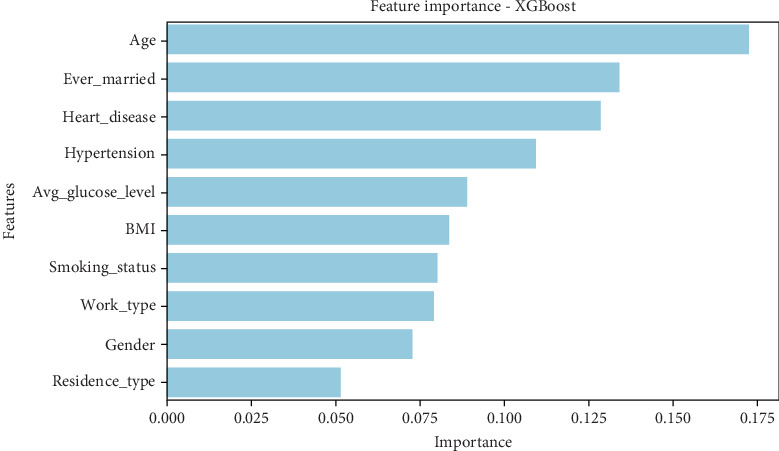
XGBoost feature importance.

This suggests that XGBoost captures chronic health conditions and demographic factors better than simpler models, and a graphical representation of the results can be seen in Figure 12.

The AdaBoost model (Table 13) highlights BMI (0.383) and age (0.344) as the top predictors, followed by average glucose level (0.183). Compared to other models, AdaBoost places far less emphasis on comorbidities like hypertension and heart disease, which are assigned near-zero importance. Work type (0.043), smoking status (0.025), and marital status (0.024) contribute slightly, but gender, residence type, and heart disease hold no weight in this model.

**Figure 13:**
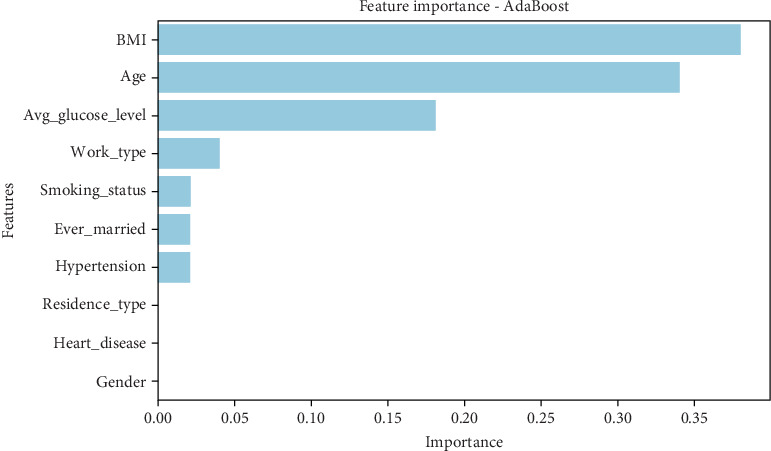
AdaBoost feature importance.

• BMI is the most significant factor in AdaBoost, surpassing age and glucose level.
• Unlike XGBoost, cardiovascular conditions (heart disease and hypertension) have minimal impact.
• Gender, residence type, and heart disease contribute nothing to predictions.
• AdaBoost emphasizes lifestyle factors (BMI, glucose, and smoking) rather than medical history, and a graphical representation of the results can be seen in Figure 13.

This suggests that AdaBoost prioritizes recent lifestyle risk factors over long-term chronic conditions when predicting stroke.

#### 5.5.1. Comparative Insights and Model Implications

The variation in feature importance across models reveals distinct patterns in how stroke risk is predicted:

1. Age and glucose levels as core predictors:
  • Age is a dominant predictor in gradient boosting, XGBoost, and AdaBoost, while glucose levels are critical in the decision tree and gradient boosting.
  • This aligns with clinical evidence that aging and high blood sugar contribute significantly to stroke risk.
2. BMI's influence varies across models:
  • AdaBoost ranks BMI as the most influential factor (0.38), while it holds moderate importance in the decision tree (0.247) and gradient boosting (0.180).
  • XGBoost ranks it much lower, suggesting that some models prioritize obesity more than others.
3. Comorbidities and medical history have a mixed impact:
  • XGBoost gives high importance to heart disease and hypertension, but AdaBoost nearly disregards them.
  • Gradient boosting and decision tree assign moderate importance to these features, indicating they are relevant but not always crucial in prediction.
4. Demographic and lifestyle factors play a minor role:
  • Smoking status has low importance across all models (0.02–0.08), indicating that smoking alone may not be a strong stroke predictor without other risk factors.
  • Work type, gender, and residence type are generally insignificant across models, showing that socioeconomic factors have a limited impact on stroke prediction.
5. Differences in model focus:
  • Decision tree and gradient boosting favor lifestyle-related factors (BMI and glucose levels).
  • XGBoost emphasizes chronic health conditions (heart disease and hypertension).
  • AdaBoost prioritizes recent lifestyle choices over long-term medical history.

The feature importance analysis reveals how different ML models weigh risk factors differently when predicting stroke. Age and glucose levels consistently appear among the most significant predictors, but their relative importance varies. BMI is highly relevant in some models (AdaBoost) but less so in others (XGBoost). Similarly, chronic conditions like heart disease and hypertension are emphasized in XGBoost but largely ignored in AdaBoost.

These findings indicate that model selection plays a crucial role in determining which risk factors are prioritized in stroke prediction. If the goal is to predict strokes based on lifestyle changes, models like AdaBoost and gradient boosting may be more useful. However, if the focus is on long-term medical conditions, XGBoost may be the better choice. Further fine-tuning of these models, incorporating feature selection, and balancing datasets could help improve overall predictive accuracy while ensuring the most critical risk factors are appropriately weighted, as presented in Table 14.

**Figure 14:**
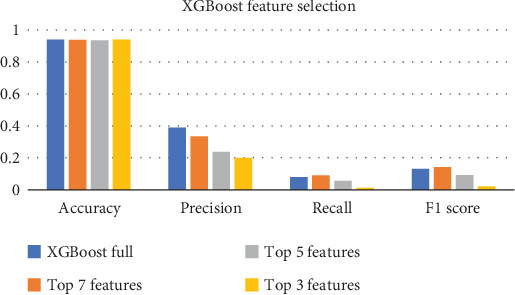
XGBoost feature selection.

Key observations:

1. Accuracy: The accuracy of the XGBoost model remains very close across all feature sets, with a slight decrease from full (0.939) to Top 7 (0.936) and further decline with Top 5 (0.934) and Top 3 (0.940). This shows that the classifier is still performing similarly across all feature sets. Notably, the accuracy does not significantly change when reducing the features from the full set down to the Top 3.
2. Precision: Precision drops as we reduce the number of features. The full dataset has a precision of 0.388, which decreases progressively as we use fewer features. This suggests that when using fewer features, the classifier may become less confident in its positive predictions, leading to more FPs. The drop-in precision from Top 7 to Top 3 is particularly significant, indicating that with fewer features, the model is less precise in identifying TP instances.
3. Recall: Recall also shows a significant decrease as we move from the full dataset to the reduced feature sets. With the Top 3 features, the recall is very low (0.011), indicating that the model is failing to identify almost all of the actual positive cases (strokes). This sharp decline in recall suggests that the Top 3 features are insufficient to accurately predict the positive class, potentially causing the model to miss a lot of TPs (FNs).
4. *F*1-score: *F*1-score, being a harmonic mean of precision and recall, also decreases with fewer features. The full dataset has the highest *F*1-score (0.130), but it drops dramatically with the Top 3 features (down to 0.021). This indicates a significant trade-off between precision and recall when reducing features, especially as recall diminishes. The results are shown in Figure 14.

• Feature importance trade-off: The full dataset provides the best balance between precision, recall, and *F*1-score, indicating that using a greater number of features allows the model to make better predictions. As you reduce the features, especially down to the Top 3 most important features, the model becomes more biased toward a particular subset of the data that may not capture the underlying patterns as well. This is reflected in the steep decline in recall and precision, leading to a lower *F*1-score.
• Feature selection impact: While accuracy remains fairly consistent, the drastic drop in recall and precision when using fewer features implies that XGBoost relies on a wider set of features to distinguish between the classes (stroke vs. no stroke). The reduction in features removes potentially crucial information that helps the model identify the positive class (stroke).
• Model's sensitivity: XGBoost seems sensitive to the number of features used in the model. While accuracy stays relatively high, the significant drop in precision and recall (especially recall) when reducing features highlights that the classifier is highly dependent on the chosen features. In particular, the model might be struggling to correctly classify stroke patients when only a small number of features are available (such as the Top 3 features).

The XGBoost model's performance across different feature sets suggests that more features are beneficial for improving the model's classification of strokes. While reducing features can decrease accuracy, the drop in recall and precision signals that the classifier's ability to correctly identify stroke cases diminishes as important features are removed.

From a clinical perspective, the identification of glucose levels, BMI, and hypertension as dominant predictors has direct implications for screening and prevention. Elevated glucose levels suggest that patients with diabetes or prediabetes should undergo routine stroke risk assessment, as poor glycemic control significantly increases vascular risk. Similarly, higher BMI values highlight the importance of integrating weight management and lifestyle modification programs into prevention initiatives, while hypertension, a well-established modifiable risk factor, reinforces the need for early detection and aggressive management of blood pressure in primary care and community health programs. Although feature importance varied across models, for example, AdaBoost emphasized BMI and age, while XGBoost highlighted heart disease and hypertension, these differences reflect algorithmic mechanisms rather than contradictions with clinical knowledge. In fact, glucose, BMI, and age consistently appeared as top predictors across methods, aligning with well-established stroke risk factors. Variability in the weight assigned to comorbidities such as heart disease and hypertension underscores the importance of considering multiple modeling approaches, suggesting that while core risk factors remain stable, the relative contribution of individual comorbidities may differ depending on patient population and data structure, consistent with findings from large cohort studies.

### 5.6. Survival Analysis

Survival analysis is a crucial statistical technique used to estimate the probability of an event occurring over time. In the context of stroke prediction, survival analysis helps determine the likelihood of patients remaining stroke-free over a given period, considering various risk factors. The Kaplan–Meier estimator and CoxPH model are commonly used methods in such studies to analyze time-to-event data. This dataset provides insights into stroke survival probabilities over different timelines, allowing researchers and healthcare professionals to assess the impact of factors such as age, glucose levels, and hypertension on stroke risk. The survival probability, initially set at 1 (100% survival), gradually decreases as time progresses, indicating the increasing likelihood of stroke occurrence among at-risk individuals. Although the Kaggle dataset is cross-sectional and does not contain time-to-event follow-up, the Kaplan–Meier and CoxPH analyses were applied here in an exploratory manner to demonstrate methodological applicability rather than to produce definitive survival estimates. It should be noted that the Kaggle dataset is cross-sectional and does not provide subject-level time-to-event or censoring information. As such, the Kaplan–Meier and CoxPH analyses presented here are exploratory demonstrations rather than true longitudinal survival models, and no formal risk-set construction or censoring was possible. It should be noted that the Kaggle dataset is cross-sectional and does not provide subject-level time-to-event or censoring information. As such, the Kaplan–Meier and CoxPH analyses presented here are exploratory demonstrations rather than true longitudinal survival models, and no formal risk-set construction or censoring was possible. It should be noted that the dataset used is cross-sectional, without longitudinal follow-up or censoring information. Therefore, the Kaplan–Meier and CoxPH analyses conducted here are exploratory and illustrative, rather than valid time-to-event survival estimates.

Understanding these patterns can aid in developing preventive strategies and improving early intervention methods for high-risk patients, as seen in Table 15.

CoxPH regression results are presented in Table 15. Hypertension showed a hazard ratio (HR) of 1.319 (95% CI: 0.973–1.789), suggesting a 31.9% increased hazard of stroke, though the CI crosses 1.0, indicating borderline significance. BMI was also significant, with HR = 1.029 (95% CI: 1.007–1.053), meaning each unit increase in BMI increases stroke hazard by ~2.9%. Elevated glucose levels showed HR = 1.003 (95% CI: 1.001–1.005), indicating higher stroke risk with rising glucose values. Heart disease, while positively associated (HR = 1.034, 95% CI: 0.728–1.468), was not statistically significant. These results highlight that hypertension, BMI, and glucose levels are clinically meaningful predictors of stroke risk.

**Figure 15:**
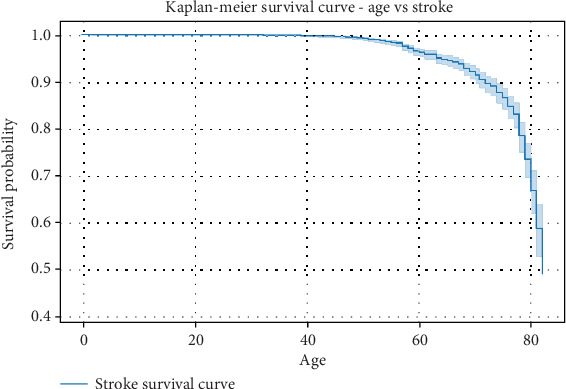
The Kaplan–Meier survival curve, age vs. stroke.

The Kaplan–Meier survival curve (Figure 15) illustrates the probability of remaining stroke-free over time. The survival probability starts at 1.0, meaning that all individuals are initially considered stroke-free. As time progresses, the survival probability gradually declines, reflecting the occurrence of stroke events.

1. Initial period (0–1.3 years)
  • During the first 1.3 years, there was no observed decrease in survival probability, indicating that no stroke events were recorded in this timeframe.
  • The survival probability remains constant at 1.0, suggesting a stable period where individuals are less likely to experience a stroke.
2. Gradual decline in survival probability (1.3–40 years)
  • The survival probability starts decreasing after 1.32 years, reaching 0.9998.
  • This slow decline suggests that stroke cases begin to emerge, though the occurrence remains relatively low in this phase.
  • Between 30–40 years, the survival probability declines more rapidly, indicating an increased number of stroke events in older age groups.
3. Notable decrease in stroke-free probability (40–60 years)
  • A significant drop in survival probability occurs beyond 40 years, where the probability reduces from 0.9976 to 0.9914.
  • This trend suggests that middle-aged individuals are more vulnerable to stroke, possibly due to the cumulative effects of risk factors such as hypertension, heart disease, or high glucose levels.
4. Sharp decline beyond 60 years
  • From 60 years onward, the survival probability starts to drop at a much higher rate, declining from 0.96 to 0.74.
  • By age 80, the probability has decreased significantly to 0.49, indicating that nearly half of the population has experienced a stroke.
  • This sharp decline in survival probability suggests that aging is a strong determinant of stroke risk, reinforcing the need for early monitoring and intervention in older populations.
5. Critical drop beyond 80 years
  • The most significant drop occurs beyond age 80, where the survival probability declines to 0.49.
  • This indicates that individuals who reach their 80s have a nearly 50% chance of experiencing a stroke, highlighting the urgent need for preventive measures in this age group.

Key insights from the stroke survival analysis:

• Younger individuals (below 40 years) have a high stroke-free probability, indicating that stroke is relatively rare in early adulthood.
• Middle-aged individuals (40–60 years) experience a moderate decline in survival probability, suggesting that risk factors start accumulating and influencing stroke incidence.
• Elderly individuals (60+ years) exhibit a rapid decrease in survival probability, emphasizing that aging is a primary risk factor for stroke.
• By age 80, the survival probability reaches nearly 50%, highlighting the importance of medical intervention and lifestyle modifications to reduce stroke risk in older adults.

The survival analysis highlights that stroke risk increases significantly with age, particularly beyond 60 years. The findings emphasize the need for early screening, lifestyle modifications, and medical interventions to mitigate risk factors such as high glucose levels, hypertension, and obesity. Future research could explore additional variables, including genetic predisposition and socioeconomic factors, to further refine stroke risk prediction models and develop targeted prevention strategies.

Reporting effect sizes with confidence intervals strengthens the interpretability of our survival analysis. For example, hypertension and BMI demonstrated clinically meaningful HRs, consistent with prior evidence that elevated blood pressure and obesity substantially increase stroke risk. The modest but significant effect of glucose levels underscores the importance of glycemic control in stroke prevention. Confidence intervals further highlight the precision and reliability of these estimates, allowing clinicians to better contextualize risk in patient care.

A key limitation of this study is that the Kaggle dataset is cross-sectional and not longitudinal, which precludes true time-to-event modeling. Consequently, the Kaplan–Meier and CoxPH analyses presented here should be regarded as illustrative rather than confirmatory, as the dataset lacks subject-level time-to-event and censoring information. This absence introduces potential prevalence and length bias, meaning that the reported HRs can only be interpreted as exploratory associations rather than valid longitudinal risk estimates. The results should not be misinterpreted as true survival probabilities but rather as illustrative associations. Future work using longitudinal clinical datasets with proper follow-up will be necessary to accurately assess survival probabilities and HRs through valid survival analysis.

### 5.7. Health Condition Analysis

The objective of this analysis is to evaluate how certain health conditions, hypertension, heart disease, and smoking status, are associated with the likelihood of experiencing a stroke. By identifying significant relationships, this study aims to provide insights into potential risk factors for stroke, which could be valuable for early intervention and prevention efforts.

To examine the impact of these health conditions on stroke occurrence, the following methods were used:

• Cross-tabulation analysis: This method helps to visually represent the distribution of stroke occurrences across different health condition categories.
• Chi-square test for independence: This statistical test assesses whether there is a significant association between each health condition and stroke occurrence. A small *p* value (typically <0.05) suggests a statistically significant relationship between the variables.

Hypertension and stroke results can be seen in Table 16.

• Among individuals with no hypertension, 183 out of 4612 had a stroke (~3.97%).
• Among those with hypertension, 66 out of 498 had a stroke (~13.25%).
• The stroke rate is significantly higher in individuals with hypertension, suggesting a strong association between hypertension and stroke risk.

Chi-square test result:

• *p* value = 1.66e − 19 (very small, statistically significant).
• Since the *p* value is much lower than 0.05, we reject the null hypothesis, confirming that hypertension is significantly associated with stroke occurrence.

Heart disease and stroke results can be seen in Table 17.

• Among individuals without heart disease, 202 out of 4834 had a stroke (~4.18%).
• Among those with heart disease, 47 out of 276 had a stroke (~17.03%).
• The stroke rate is much higher in individuals with heart disease, suggesting a significant correlation between heart disease and stroke occurrence, as seen in Table 17.

Chi-square test result:

• *p* value = 2.09e − 21 (highly significant).
• This result confirms that heart disease is significantly associated with stroke risk.

Smoking status and stroke results can be seen in Table 18.

• The highest stroke rate is among individuals who formerly smoked (70 out of 885, ~7.91%).
• Those who currently smoke also have an elevated stroke rate (42 out of 789, ~5.32%).
• The lowest stroke rate is among those with an unknown smoking status (47 out of 1544, ~3.04%).
• The stroke rate among never smokers is 4.76%, which is lower than former smokers but still above the unknown group.
• This suggests that past smoking may have a lasting impact on stroke risk.

Hypertension and heart disease are strongly associated with stroke occurrence, with both conditions exhibiting significantly higher stroke rates. This relationship is statistically confirmed through chi-square test results, which yielded very small *p* values, indicating a robust correlation. Additionally, smoking status plays a role in stroke likelihood, with former smokers showing the highest stroke rate, suggesting that the effects of past smoking behavior continue to influence long-term health outcomes. Based on these findings, public health strategies should prioritize the management and prevention of hypertension and heart disease, alongside promoting effective smoking cessation programs, to significantly reduce stroke risk in the population.

### 5.8. Clustering Analysis

In the context of the clustering analysis and the visualizations using PCA and t-SNE, the components refer to the transformed features or dimensions that are generated during the dimensionality reduction process. These components are essentially new axes that represent the most important information in the original data, but in fewer dimensions (usually two dimensions for visualization). It is important to note that PCA and t-SNE were applied in this study for exploratory visualization rather than validated clustering. Future studies should include quantitative cluster validity indices (e.g., silhouette score and the Davies–Bouldin index) to confirm the robustness of subgroup structures.

Table 19 presents the standardized mean values of key health-related features, age, average glucose level, BMI, and smoking status across three distinct clusters identified through unsupervised clustering analysis. These clusters were derived using dimensionality reduction techniques such as PCA and t-SNE, followed by *K*-means clustering. The values have been normalized to facilitate comparison, with each feature's average in a cluster indicating its relative deviation from the dataset's overall mean. This analysis aims to uncover distinct patient profiles and highlight potential high-risk groups for stroke based on shared characteristics. Notably, Cluster 2 exhibits the highest positive deviations in age and glucose levels, suggesting a group with elevated stroke risk.

#### 5.8.1. PCA

PCA is a technique used for reducing the dimensionality of the data while retaining as much variance (information) as possible. It does this by creating principal components, which are the new axes that capture the maximum variance in the data.

PCA components: These are the transformed dimensions after applying PCA.

• The first component (PCA Component 1) captures the largest variance (spread of data) in the dataset.
• The second component (PCA Component 2) captures the second-largest variance and so on.

The PCA components are the new coordinates for each data point after reducing the dataset from high-dimensional space (e.g., many features like age, glucose level, and smoking status) to just two dimensions (for plotting).

**Figure 16:**
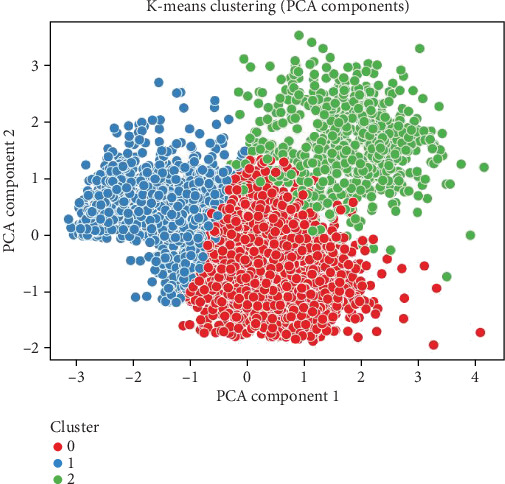
*K*-means clustering (PCA components).

The scatter plot (Figure 16) shows how the data points group together based on the features, and the clusters are represented by different colors.

PCA Component 1 is plotted on the *x*-axis, and PCA Component 2 is plotted on the *y*-axis in the 2D graph.

Example: If you had a dataset with three features (age, glucose level, and BMI), PCA would reduce it to two components. The new axes (PCA1 and PCA2) are linear combinations of the original features, and the scatter plot shows how data points are spread along these axes.

#### 5.8.2. t-SNE

t-SNE is another dimensionality reduction technique, but it works differently from PCA. t-SNE is particularly useful for visualizing high-dimensional data by maintaining the pairwise similarity of data points in the lower dimensional space (2D or 3D). It does this by mapping similar data points close together and dissimilar ones farther apart.

t-SNE components: These are the coordinates of the data points after t-SNE has reduced the data to two dimensions.

• t-SNE Component 1 is the first coordinate (plotted on the *x*-axis).
• t-SNE Component 2 is the second coordinate (plotted on the *y*-axis).

**Figure 17:**
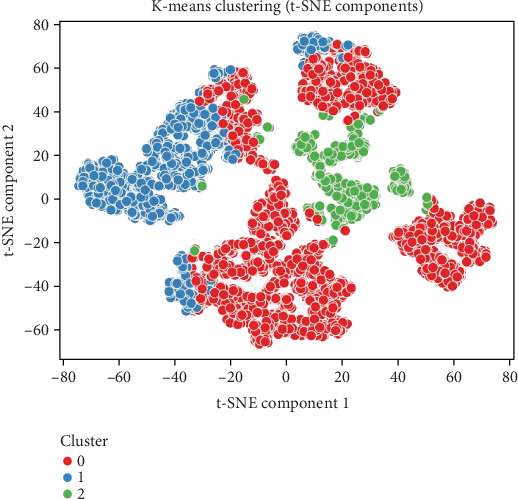
*K*-means clustering (t-SNE components).

In the graphs:

The scatter plot (Figure 17) shows how the data points are grouped based on their pairwise similarities. Points that are similar (in terms of the original high-dimensional features) will appear close to each other in the 2D t-SNE plot, while points that are dissimilar will be farther apart.

The clusters in the t-SNE plot may not necessarily align with the axes of the plot, as t-SNE focuses on maintaining local structure rather than global structure like PCA.

Example: If you applied t-SNE to the same dataset (age, glucose level, and BMI), t-SNE would try to preserve the relationships between the data points in the lower-dimensional space so that similar points appear closer to each other in the t-SNE 2D plot.

• PCA components: Represent directions in the data that capture the maximum variance. They are linear combinations of the original features.
• t-SNE components: Represent a projection of the data into two dimensions, trying to preserve the local structure and relationships between points. These components do not have a straightforward interpretation like PCA components but are useful for visualizing complex data.

In both clustering graphs:

The *x*-axis and *y*-axis represent the first two components (PCA or t-SNE) after dimensionality reduction.

Clusters are colored according to the group assignment from *K*-means clustering.

These plots help in visualizing the clustering of data based on the reduced dimensions, making it easier to understand how data points group together according to their features.

To analyze the results you provided, we first need to understand that these values appear to be normalized or standardized values for the features in the dataset. Each feature (e.g., age, avg_glucose_level, BMI, and smoking_status) has been transformed in such a way that the data is centered around 0 with a certain scale (most likely with 0 mean and unit variance, if standardization was used).

#### 5.8.3. Understanding the Values

Each row represents an individual data point, and each column represents one of the features (age, glucose level, BMI, and smoking status) that have been standardized. Let us break this down for each feature:

1. Age:

In the first row, the value for age is 0.3193. This indicates that the individual's age is slightly above the mean age of the dataset (since it is positive).

In the second row, the age is −1.1114, indicating that this individual is below the average age of the dataset (since it is negative).

In the third row, the age is 0.7760, which means this individual's age is above the mean, but not as much as the first individual.

2. Average glucose level:

In the first row, the glucose level is −0.3656. This indicates that this individual has a glucose level slightly below the average glucose level.

In the second row, the glucose level is −0.2760, which is also slightly below average, but less so than the first individual.

In the third row, the glucose level is 2.2042, which is much higher than average. This suggests that the third individual has a significantly higher-than-average glucose level.

3. BMI

In the first row, the BMI is 0.2363, indicating that this individual's BMI is slightly above the average BMI.

In the second row, the BMI is −0.8255, meaning this individual's BMI is well below the average.

In the third row, the BMI is 0.5803, showing that the individual has a higher BMI than the average.

4. Smoking status:

In the first row, the smoking status is 0.3954, which likely represents a “positive” status (e.g., smoker, or a value that indicates some kind of smoker category).

In the second row, the smoking status is −0.9978, which could represent a “negative” status (e.g., nonsmoker).

In the third row, the smoking status is 0.2050, which is somewhere in between, possibly indicating that the individual has a low probability of being a smoker (or another factor).

#### 5.8.4. Statistical Significance and Interpretation

• Standardization: Since these features are standardized, you can now analyze their relationship with each other and their impact on the outcome variable (e.g., stroke risk, if that is the target variable). Values closer to 0 indicate that the individual is close to the average of the dataset for that feature, while values further from 0 indicate more extreme values (either higher or lower than average).
• Comparing the features: You can also explore how each feature (age, glucose level, BMI, and smoking status) contributes to the likelihood of stroke (or any other outcome variable). For example, based on these standardized values:
  o. Age: A positive value means older individuals are more likely to be above average. Age often has a direct correlation with health outcomes like stroke, so you may expect older individuals to have a higher risk of stroke.
  o. Glucose level: The third individual, with a significantly higher glucose level, could be at a higher risk of conditions such as diabetes or stroke, which is reflected in the high positive value (2.2042).
  o. BMI: This could show that individuals with a higher BMI (first and third individuals) might have a higher likelihood of developing health problems such as stroke.
  o. Smoking status: A higher value might indicate a higher likelihood of being a smoker, which is often linked to an increased risk of stroke and other health problems.

Our clustering results should be interpreted cautiously, as they were not validated using internal indices such as silhouette or the Davies–Bouldin scores. Consequently, the clinical interpretation of clusters remains speculative. Future work should include both quantitative validation and external dataset confirmation to ensure subgroup definitions are robust and clinically meaningful.

## 6. Conclusion

This study provides a comprehensive, multifaceted analysis of stroke risk by integrating statistical methods, ML classification, clustering techniques, and survival analysis to uncover key predictive factors associated with stroke occurrence. The dataset, comprising 5110 observations, highlights critical demographic, medical, and lifestyle attributes that contribute to stroke risk. Descriptive statistics indicate that age, glucose levels, BMI, hypertension, and heart disease are among the most influential variables, with older individuals, elevated glucose levels, and higher BMI being more prone to stroke. Correlation analysis further confirms that age is positively associated with hypertension and heart disease, while glucose levels also show a moderate correlation with age, indicating that older individuals tend to have higher glucose levels, potentially increasing stroke susceptibility. Health condition analysis reveals a significant relationship between stroke and underlying medical conditions, with hypertensive individuals having a stroke rate of 13.25% compared to 3.97% among nonhypertensive individuals and those with heart disease exhibiting a stroke rate of 17.03%, significantly higher than those without heart disease. Additionally, former smokers (7.91%) and current smokers (5.32%) show an elevated stroke risk, emphasizing the long-term impact of smoking on cardiovascular health. Clustering and dimensionality reduction techniques using PCA and t-SNE effectively identify high-risk groups, with one cluster exhibiting significantly higher glucose levels and older age, suggesting an elevated likelihood of stroke. EDA further highlights that hypertensive individuals have a significantly higher mean age (62.24 years) compared to nonhypertensive individuals (41.17 years), while stroke-positive individuals exhibit a markedly higher mean glucose level (132.54 mg/dL) compared to nonstroke individuals (104.79 mg/dL), reinforcing the role of metabolic disorders in stroke occurrence. Similarly, stroke-positive individuals have a slightly higher mean BMI (30.47 kg/m^2^) compared to nonstroke individuals (28.82 kg/m^2^), indicating that obesity may contribute to stroke risk, although not as significantly as glucose levels. ML classification models provide further insights into stroke prediction, with gradient boosting, XGBoost, and CatBoost achieving high accuracy (>94%) but suffering from low recall, making them less effective in clinical applications where missing stroke cases can have severe consequences. In contrast, naïve Bayes achieves the highest recall (0.404), detecting more stroke cases but at the cost of increased FPs. Among these models, XGBoost demonstrates the best balance between precision and recall, making it a viable candidate for predictive stroke modeling. Feature importance analysis across various classifiers indicates that glucose levels, BMI, and age are the most critical stroke predictors, with the decision tree ranking glucose levels as the most significant factor, while gradient boosting prioritizes age and glucose levels equally. XGBoost, however, assigns higher importance to heart disease and hypertension, suggesting that pre-existing cardiovascular conditions play a crucial role in stroke prediction. Survival analysis using the Kaplan–Meier estimator confirms that stroke risk increases significantly with age, with the probability of remaining stroke-free sharply declining beyond 60 years. CoxPH regression further supports these findings, indicating that hypertension increases stroke risk by 31.9%, glucose levels have a minor but statistically significant impact, and BMI also contributes moderately to stroke occurrence. The combination of statistical analysis, clustering, ML classification, and survival modeling provides a holistic approach to understanding stroke risk, reinforcing the importance of early detection, lifestyle modifications, and targeted medical interventions to mitigate risk factors and improve healthcare outcomes.

This study offers a comprehensive, multidimensional analysis of stroke risk by integrating statistical methods, ML classification, clustering, and survival analysis to extract valuable insights. Key findings highlight that age, glucose levels, BMI, hypertension, and heart disease are the most influential predictors of stroke, with smoking, especially among former smokers, further increasing risk. While ML models such as XGBoost and gradient boosting demonstrate high accuracy, their recall rates need enhancement for clinical reliability. Survival analysis reinforces that stroke risk significantly escalates after age 60, and feature selection plays a critical role in model performance, particularly emphasizing the importance of glucose levels and hypertension. Future research should focus on improving recall through techniques like SMOTE to address class imbalance, exploring deep learning models for enhanced predictive power, incorporating broader risk factors such as lifestyle habits and medication use, and developing real-time risk assessment tools to support early clinical intervention. This data-driven approach underscores the importance of early detection, preventive healthcare strategies, and optimized predictive modeling to improve stroke outcomes. Another limitation of this study is that all analyses were conducted using the publicly available Kaggle stroke dataset. While this dataset provides a valuable benchmark for method development, it may not capture population-specific variations or the full heterogeneity of stroke risk factors. Future work should focus on validating these models with independent datasets, ideally from EHRs or prospective clinical studies. Such external validation would enhance generalizability, improve confidence in the models' clinical utility, and allow for tailoring to local populations.

## Figures and Tables

**Table 1:** Summary statistics of key variables.

	**Ever married**	**Work type**	**Residence type**	**Avg glucose level**	**BMI**	**Smoking status**	**Stroke**
Count	5110	5110	5110	5110	4909	5110	5110
Unique	2	5	2			4	
Top	Yes	Private	Urban			Never smoked	
Freq	3353	2925	2596			1892	
Mean				106.1477	28.89324		0.048728
Std				45.28356	7.854067		0.21532
Min				55.12	10.3		0
25%				77.245	23.5		0
50%				91.885	28.1		0
75%				114.09	33.1		0
Max				271.74	97.6		1
	**ID**	**Gender**	**Age**	**Hyper.**	**Heart disease**		
Count	5110	5110	5110	5110	5110		
Unique		3					
Top		Female					
Freq		2994					
Mean	36,517.83		43.22661	0.097456	0.054012		
Std	21,161.72		22.61265	0.296607	0.226063		
Min	67		0.08	0	0		
25%	17,741.25		25	0	0		
50%	36,932		45	0	0		
75%	54,682		61	0	0		
Max	72,940		82	1	1		

**Table 2:** Attribute correlation.

	**Age**	**Avg_glucose_level**	**BMI**	**Hypertension**	**Heart_disease**
Age	1	0.238171	0.333398	0.276398	0.263796
Avg_glucose_level	0.238171	1	0.175502	0.174474	0.161857
BMI	0.333398	0.175502	1	0.167811	0.041357
Hypertension	0.276398	0.174474	0.167811	1	0.108306
Heart_disease	0.263796	0.161857	0.041357	0.108306	1

**Table 3:** Age, average glucose, and BMI results.

	**Age**	**Avg_glucose_level**	**BMI**
Count	5110	5110	4909
Mean	43.22661	106.1477	28.89324
Std	22.61265	45.28356	7.854067
Min	0.08	55.12	10.3
25%	25	77.245	23.5
50%	45	91.885	28.1
75%	61	114.09	33.1
Max	82	271.74	97.6

**Table 4:** Age vs. stroke boxplot.

**Hypertension**	**Count**	**Mean**	**Std**	**Min**	**25%**	**50%**	**75%**	**Max**
0	4612	41.17303	22.40497	0.08	23	42	58	82
1	498	62.24498	14.0548	17	52.25	63	74	82

**Table 5:** Average glucose vs. stroke boxplot.

**Stroke**	**Count**	**Mean**	**Std**	**Min**	**25%**	**50%**	**75%**	**Max**
0	4861	104.7955	43.84607	55.12	77.12	91.47	112.83	267.76
1	249	132.5447	61.92106	56.11	79.79	105.22	196.71	271.74

**Table 6:** BMI vs. stroke boxplot.

**Stroke**	**Count**	**Mean**	**Std**	**Min**	**25%**	**50%**	**75%**	**Max**
0	4700	28.82306	7.908287	10.3	23.4	28	33.1	97.6
1	209	30.47129	6.329452	16.9	26.4	29.7	33.7	56.6

**Table 7:** Classification results.

**Model**	**Accuracy**	**Precision**	**Recall**	**F**1** -score**	**AUROC**
Decision tree	0.903	0.196	0.213	0.204	0.580
Gradient boosting	0.943	1.000	0.022	0.044	0.834
KNN	0.941	0.250	0.011	0.022	0.612
Naïve Bayes	0.865	0.189	0.404	0.258	0.814
XGBoost	0.939	0.389	0.079	0.131	0.804
AdaBoost	0.939	0.250	0.022	0.041	0.819
CatBoost	0.942	0.500	0.034	0.063	0.826

**Table 8:** Comparison table using the Kaggle stroke dataset, with 5110 samples.

**Study (author, year)**	**Stroke cases/prevalence**	**Methods used**	**Reported metrics**	**Notes**
Chakraborty et al., 2024 [15]	249 (~4.9%)	Stacking ensemble (random forest + decision tree + *K*-nearest neighbors), with PCA for feature reduction; oversampling of minority class	Accuracy ~98.6%	High accuracy, but recall/sensitivity not clearly differentiated in some reports; class imbalance addressed via oversampling; demonstrates potential with PCA + stacking
Present study (2025)	249 (~4.9%)	LR, RF, XGBoost, SVM, and AdaBoost	AUROC ~0.84, *F*1-score ~0.72, and sensitivity ~0.74	Focus on interpretability, feature importance, and clinical relevance under class imbalance

**Table 9:** Confusion matrix results (normalized by the true class count) rather than raw counts.

**Model**	**TP**	**TN**	**FP**	**FN**
Decision tree	1366	78	70	19
Gradient boosting	1444	0	87	2
KNN	1441	3	88	1
Naïve Bayes	1290	154	53	36
XGBoost	1433	11	82	7
AdaBoost	1438	6	87	2
CatBoost	1441	3	86	3

**Table 10:** Decision tree feature importance.

**Feature**	**Importance**
Avg_glucose_level	0.333
BMI	0.247
Age	0.199
Smoking_status	0.065
Hypertension	0.056
Work_type	0.047
Residence_type	0.016
Gender	0.016
Heart_disease	0.014
Ever_married	0.007

**Table 11:** Gradient boosting feature importance.

**Feature**	**Importance**
Age	0.374
Avg_glucose_level	0.371
BMI	0.180
Smoking_status	0.019
Work_type	0.018
Heart_disease	0.016
Hypertension	0.009
Ever_married	0.008
Residence_type	0.004
Gender	0.000

**Table 12:** XGBoost feature importance.

**Feature**	**Importance**
Age	0.173
Ever_married	0.134
Heart_disease	0.128
Hypertension	0.109
Avg_glucose_level	0.090
BMI	0.084
Smoking_status	0.080
Work_type	0.079
Gender	0.073
Residence_type	0.052

**Table 13:** AdaBoost feature importance.

**Feature**	**Importance**
BMI	0.383
Age	0.344
Avg_glucose_level	0.183
Work_type	0.043
Smoking_status	0.025
Ever_married	0.024
Hypertension	0.021
Residence_type	0
Heart_disease	0
Gender	0

**Table 14:** Feature importance analysis when selecting a certain number of features using the XGBoost classifier.

**Feature set**	**Accuracy**	**Precision**	**Recall**	**F**1** -score**
XGBoost full	0.939	0.388	0.078	0.130
Top 7 features	0.936	0.333	0.089	0.141
Top 5 features	0.934	0.238	0.056	0.090
Top 3 features	0.940	0.200	0.011	0.021

**Table 15:** CoxPH regression.

**Covariate**	**Coef**	**Exp(coef)**	**Se(coef)**	**Coef lower 95%**	**Coef upper 95%**	**Exp(coef) lower 95%**	**Exp(coef) upper 95%**
Hypertension	0.277	1.319	0.155	−0.028	0.582	0.973	1.789
Heart_disease	0.033	1.034	0.179	−0.317	0.384	0.728	1.468
Avg_glucose_level	0.003	1.003	0.001	0.001	0.005	1.001	1.005
BMI	0.029	1.029	0.011	0.007	0.051	1.007	1.053
Covariate	cmp to	*z*	*p*	−log2(*p*)			
Hypertension	0.000	1.783	0.075	3.744			
Heart_disease	0.000	0.186	0.853	0.230			
Avg_glucose_level	0.000	2.453	0.014	6.141			
BMI	0.000	2.542	0.011	6.501			

**Table 16:** Hypertension and stroke.

**Hypertension**	**No stroke**	**Stroke**	**Total**
No	4429	183	4612
Yes	432	66	498
Total	4861	249	5110

**Table 17:** Heart disease and stroke.

**Heart disease**	**No stroke**	**Stroke**	**Total**
No	4632	202	4834
Yes	229	47	276
Total	4861	249	5110

**Table 18:** Smoking status and stroke.

**Smoking status**	**No stroke**	**Stroke**	**Total**
Unknown	1497	47	1544
Formerly smoked	815	70	885
Never smoked	1802	90	1892
Smokes	747	42	789
Total	4861	249	5110

**Table 19:** Standardized feature averages across clusters identified by clustering analysis.

**Cluster**	**Age**	**Avg_glucose_level**	**BMI**	**Smoking_status**
0	0.319	−0.366	0.236	0.395
1	−1.111	−0.276	−0.826	−0.998
2	0.776	2.204	0.580	0.205

## Data Availability

For the purpose of this study, the stroke dataset is provided by https://www.kaggle.com/datasets/fedesoriano/stroke-prediction-dataset and is used because it is widely used by the research community and is publicly available.
